# Supplementary figures and images for: Nucleosome conformation dictates the histone code
Source: eLife. 2024 Feb 6;13:e78866. doi: 10.7554/eLife.78866 (PMC10876215; doi:10.7554/eLife.78866)

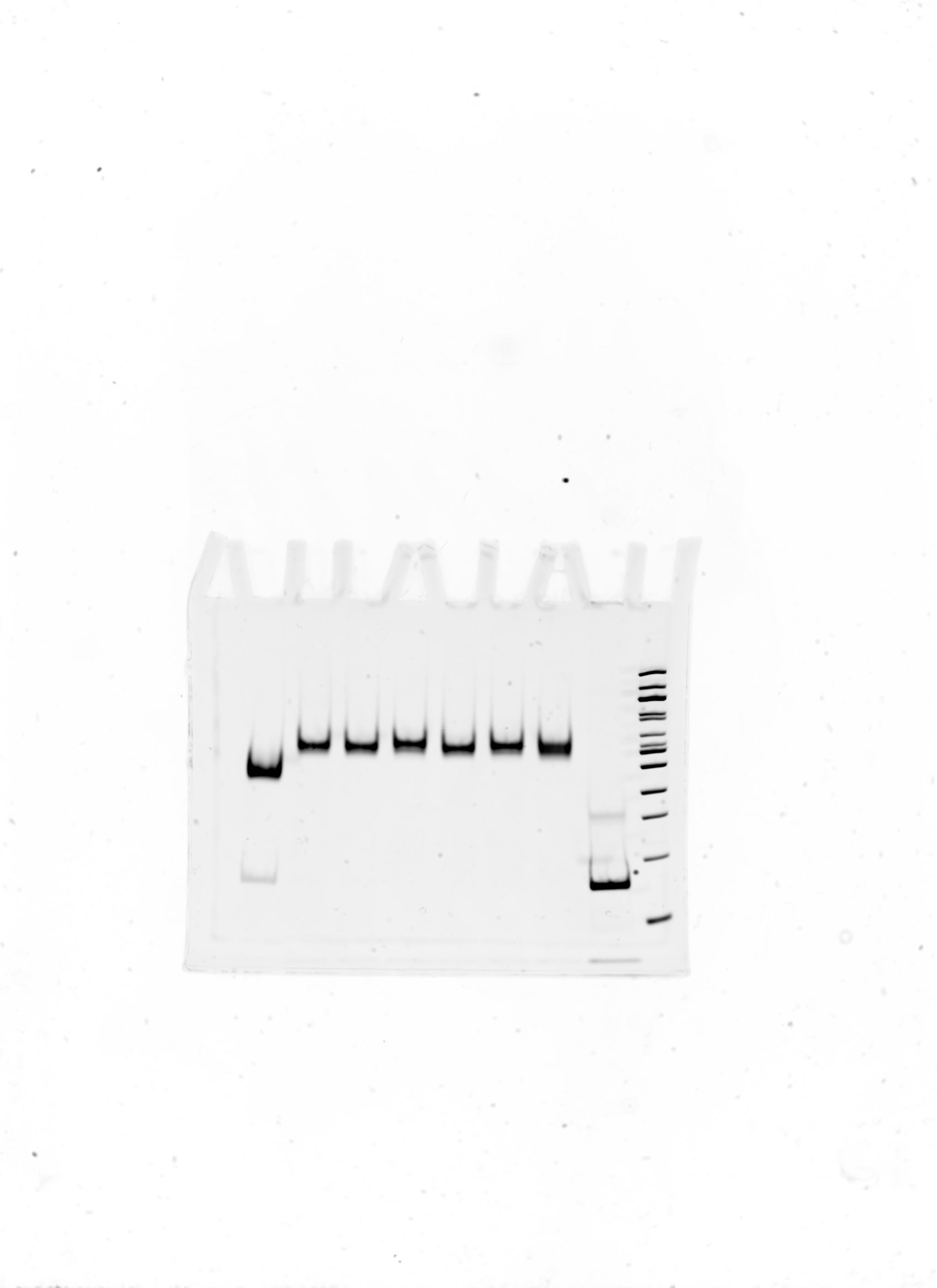

Supplement: Figure 1—figure supplement 2—source data 1. [file elife-78866-fig1-figsupp2-data1.zip › Fig1_Supplement 2_SourceData1.jpg]

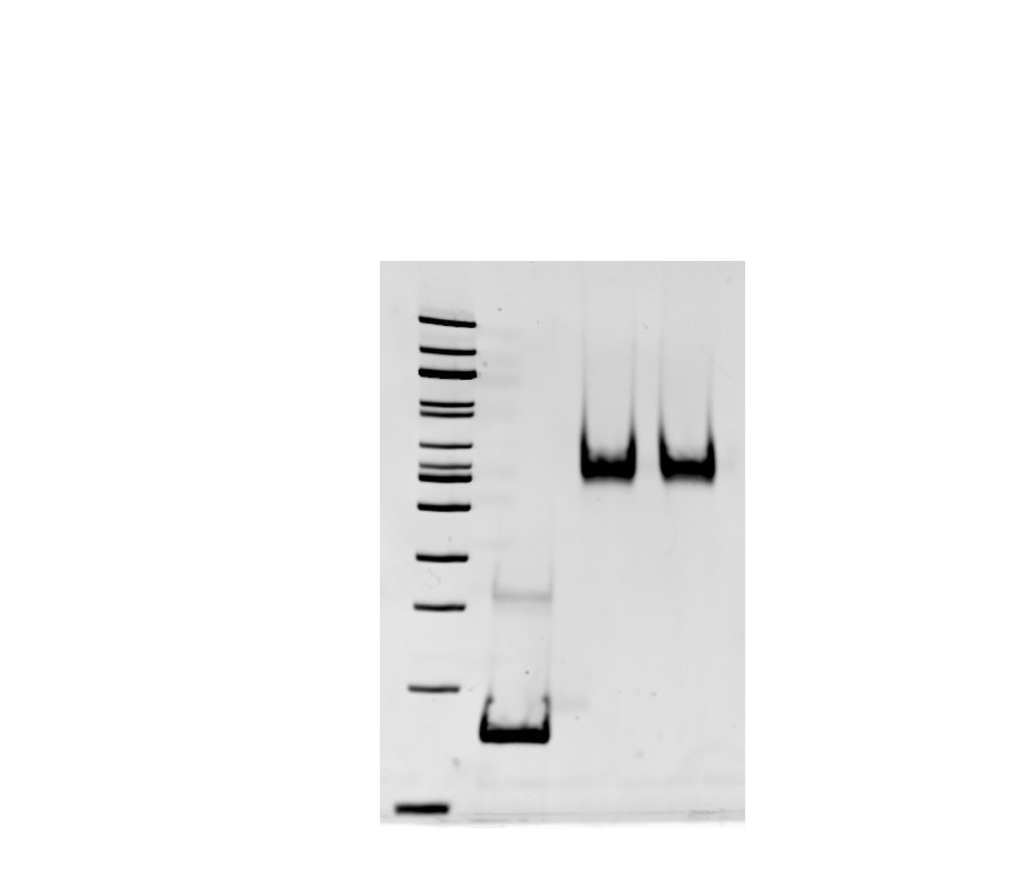

Supplement: Figure 1—figure supplement 2—source data 2. [file elife-78866-fig1-figsupp2-data2.zip › Fig1_Supplement 2_SourceData2.png]

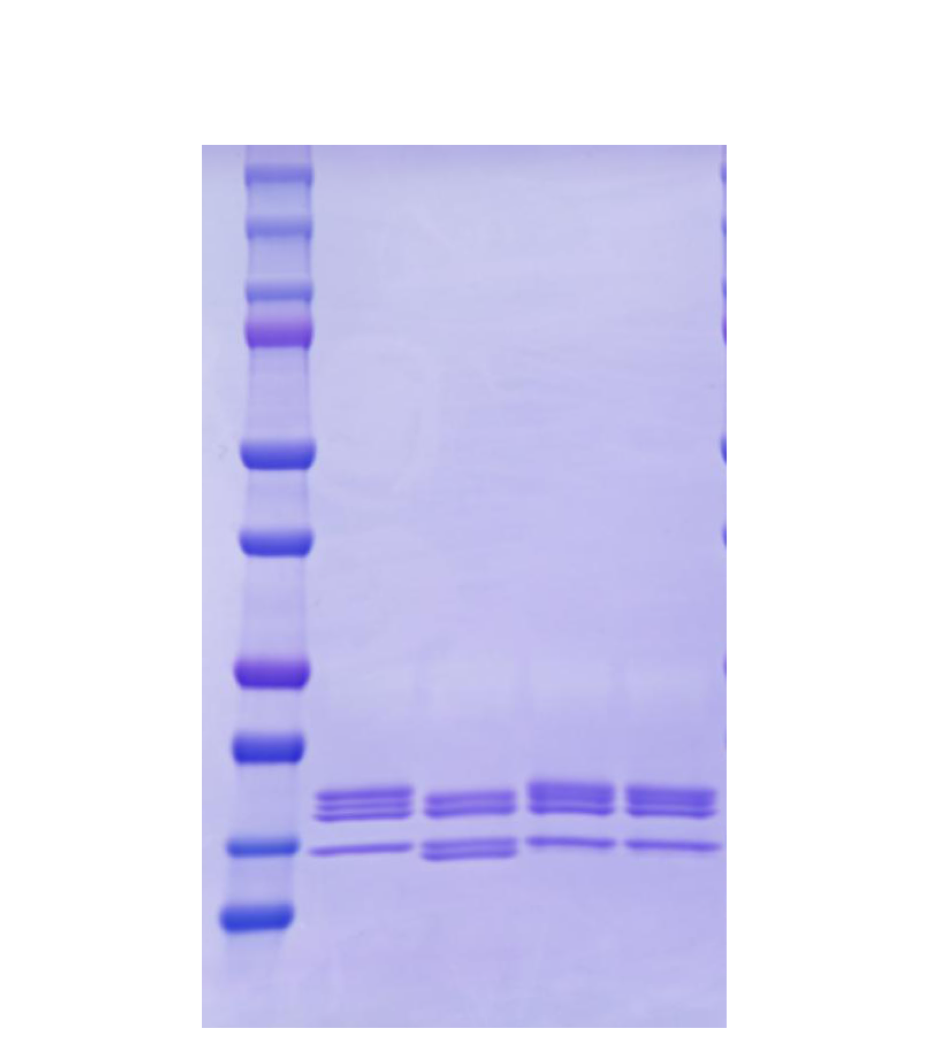

Supplement: Figure 1—figure supplement 2—source data 3. [file elife-78866-fig1-figsupp2-data3.zip › Fig1_Supplement 2_SourceData3.png]

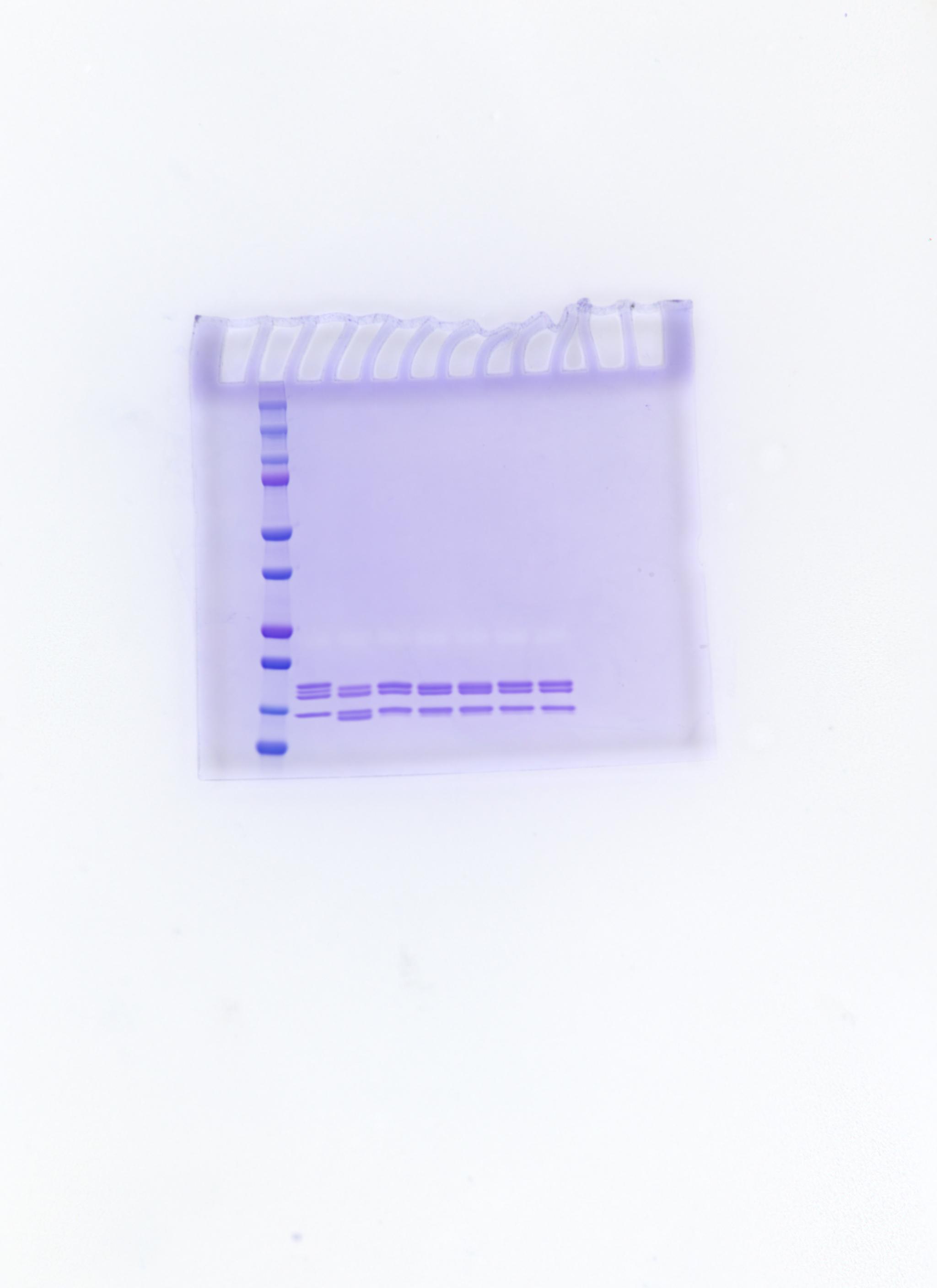

Supplement: Figure 1—figure supplement 2—source data 4. [file elife-78866-fig1-figsupp2-data4.zip › Fig1_Supplement 2_SourceData4.jpg]

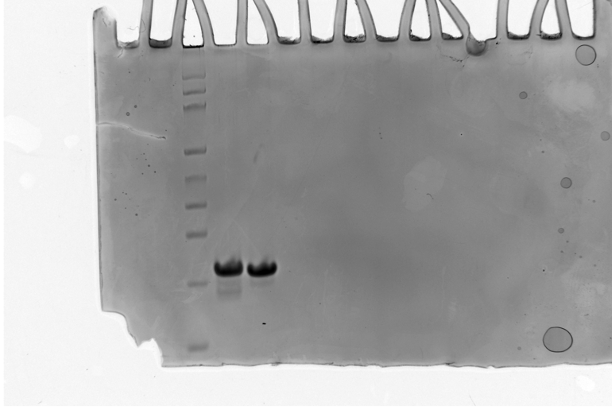

Supplement: Figure 1—figure supplement 2—source data 5. [file elife-78866-fig1-figsupp2-data5.zip › Fig1_Supplement 2_SourceData5.png]

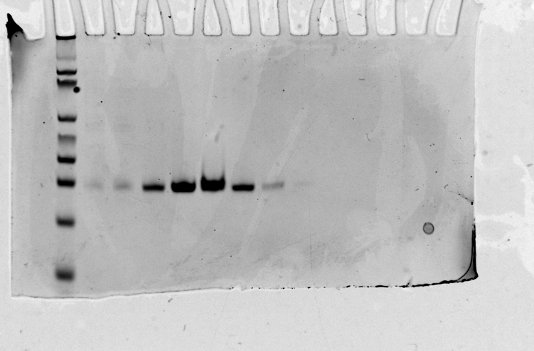

Supplement: Figure 1—figure supplement 2—source data 6. [file elife-78866-fig1-figsupp2-data6.zip › Fig1_Supplement 2_SourceData6.png]

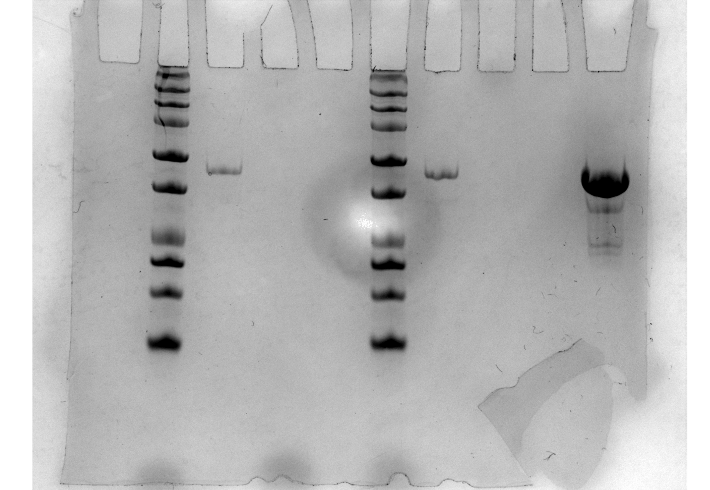

Supplement: Figure 1—figure supplement 2—source data 7. [file elife-78866-fig1-figsupp2-data7.zip › Fig1_Supplement 2_SourceData7.png]

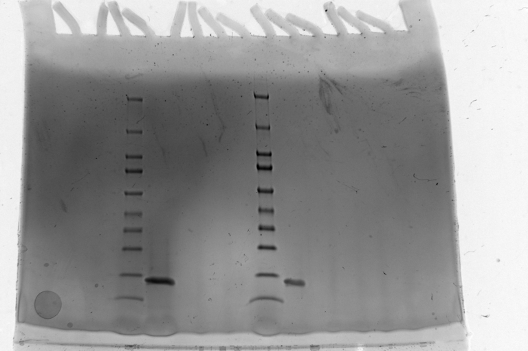

Supplement: Figure 1—figure supplement 2—source data 8. [file elife-78866-fig1-figsupp2-data8.zip › Fig1_Supplement 2_SourceData8.png]

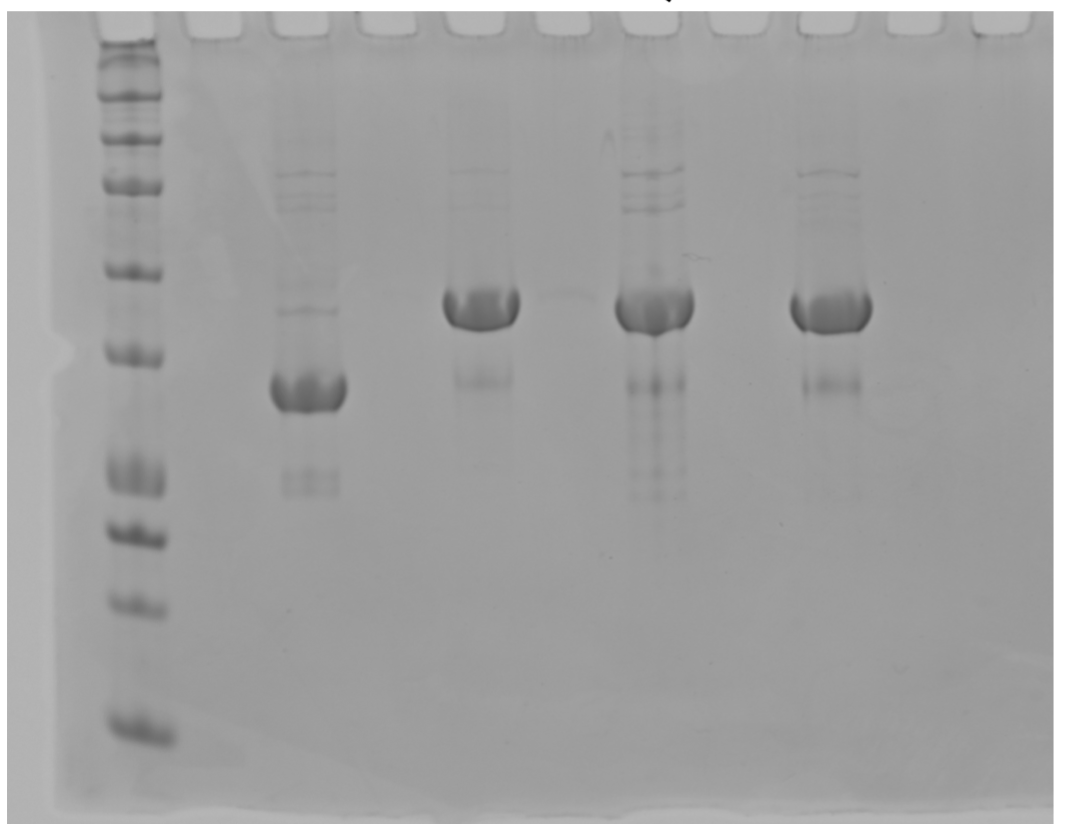

Supplement: Figure 1—figure supplement 2—source data 9. [file elife-78866-fig1-figsupp2-data9.zip › Fig1_Supplement 2_SourceData9.png]

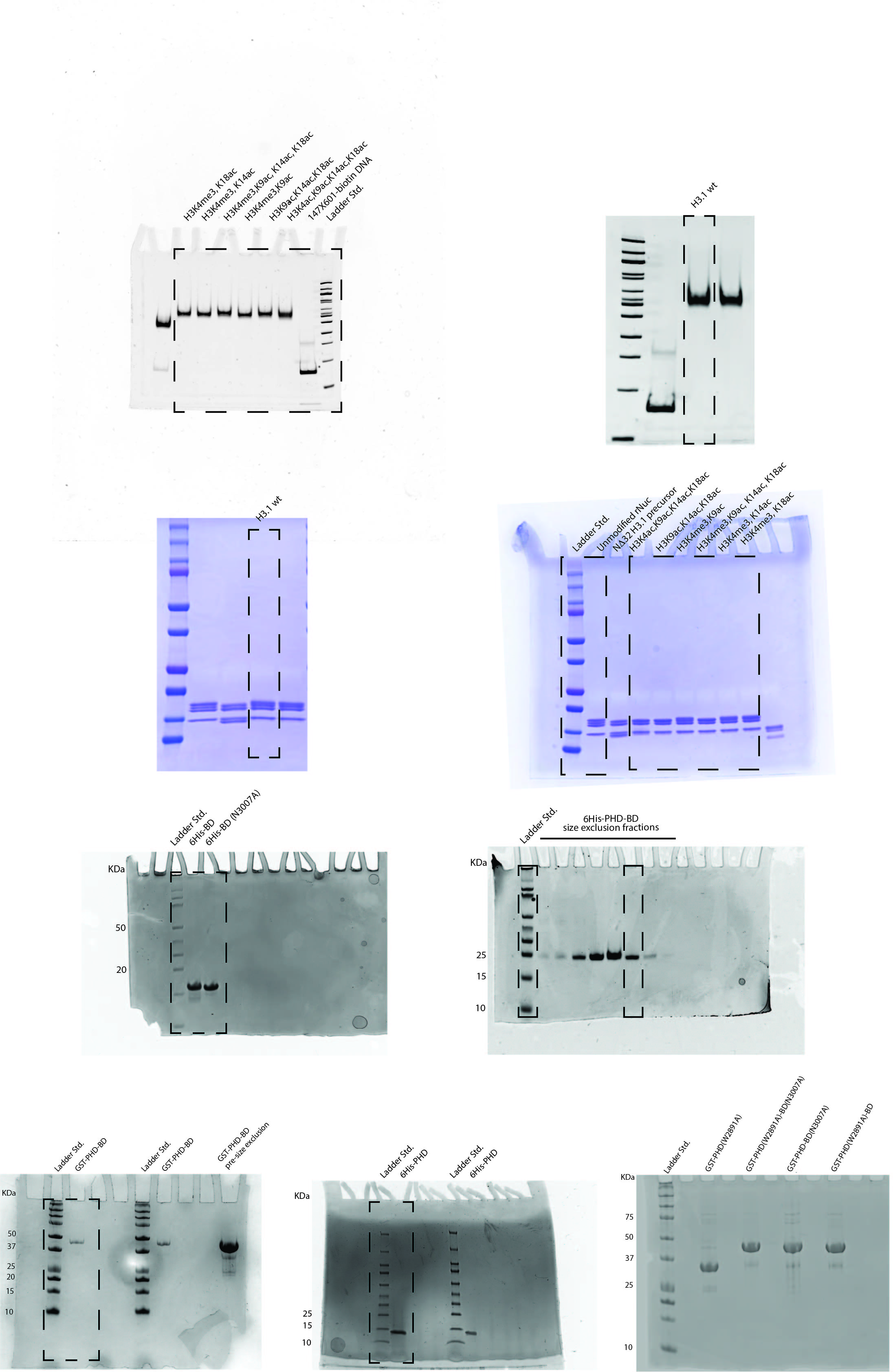

Supplement: Figure 1—figure supplement 2—source data 10. [file elife-78866-fig1-figsupp2-data10.zip › Fig1_Supplement 2_SourceData10.jpg]

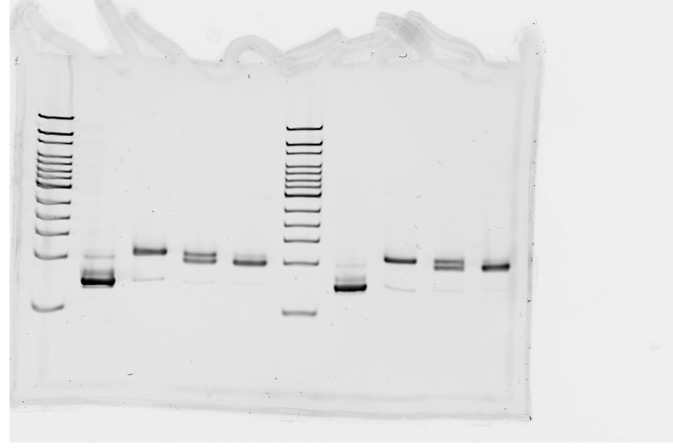

Supplement: Figure 4—figure supplement 1—source data 1. [file elife-78866-fig4-figsupp1-data1.zip › Fig4_Supplement1_SourceData1.jpg]

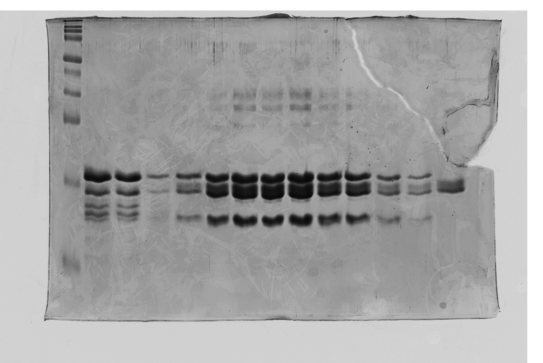

Supplement: Figure 4—figure supplement 1—source data 2. [file elife-78866-fig4-figsupp1-data2.zip › Fig4_Supplement1_SourceData2.png]

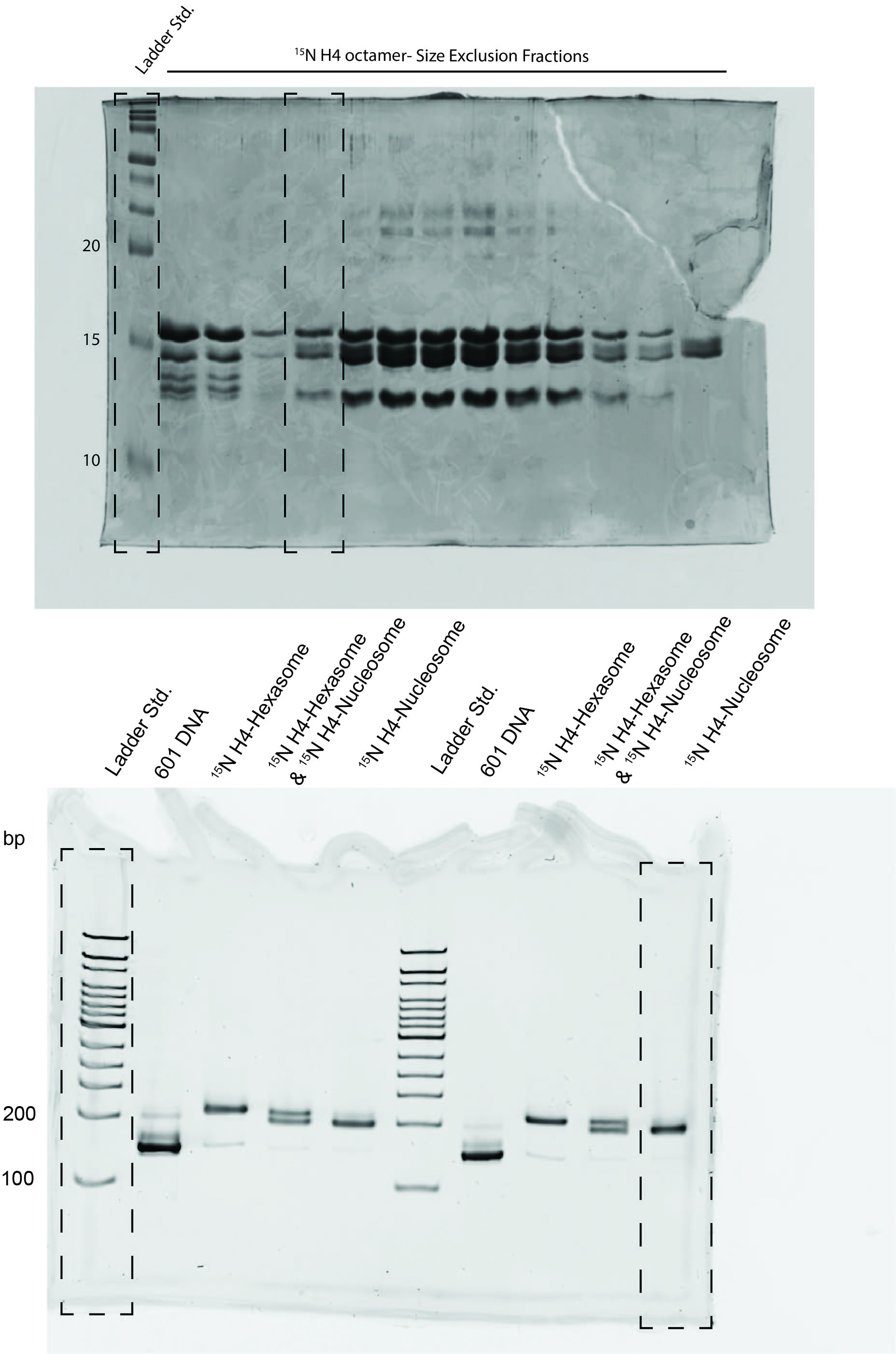

Supplement: Figure 4—figure supplement 1—source data 3. [file elife-78866-fig4-figsupp1-data3.zip › Fig4_Supplement1_SourceData3.jpg]

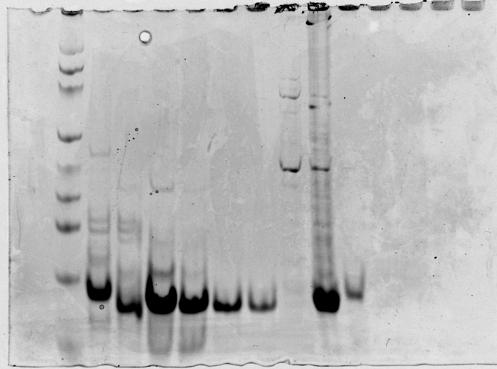

Supplement: Figure 4—figure supplement 3—source data 1. [file elife-78866-fig4-figsupp3-data1.zip › Fig4_Supplement3_SourceData1.png]
